# Deficiency in p38β MAPK Fails to Inhibit Cytokine Production or Protect Neurons against Inflammatory Insult in In Vitro and In Vivo Mouse Models

**DOI:** 10.1371/journal.pone.0056852

**Published:** 2013-02-15

**Authors:** Bin Xing, Adam D. Bachstetter, Linda J. Van Eldik

**Affiliations:** 1 Sanders-Brown Center on Aging, University of Kentucky, Lexington, Kentucky, United States of America; 2 Department of Anatomy and Neurobiology, College of Medicine, University of Kentucky, Lexington, Kentucky, United States of America; Emory University, United States of America

## Abstract

The p38 MAPK pathway plays a key role in regulating the production of proinflammatory cytokines, such as TNFα and IL-1β, in peripheral inflammatory disorders. There are four major isoforms of p38 MAPK (p38α, β, δ, γ), with p38α and p38β the targets of most p38 MAPK inhibitor drugs. Our previous studies demonstrated that the p38α MAPK isoform is an important contributor to stressor-induced proinflammatory cytokine up-regulation and neurotoxicity in the brain. However, the potential role of the p38β MAPK isoform in CNS proinflammatory cytokine overproduction and neurotoxicity is poorly understood. In the current studies, we used primary microglia from wild type (WT) and p38β knockout (KO) mice in co-culture with WT neurons, and measured proinflammatory cytokines and neuron death after LPS insult. We also measured neuroinflammatory responses *in vivo* in WT and p38β KO mice after administration of LPS by intraperitoneal or intracerebroventricular injection. WT and p38β KO microglia/neuron co-cultures showed similar levels of TNFα and IL-1β production in response to LPS treatment, and no differences in LPS-induced neurotoxicity. The *in vitro* results were confirmed *in vivo*, where levels of TNFα and IL-1β in the CNS were not significantly different between WT or p38β KO mice after LPS insult. Our results suggest that, similar to peripheral inflammation, p38α is critical but p38β MAPK is dispensable in the brain in regards to proinflammatory cytokine production and neurotoxicity induced by LPS inflammatory insult.

## Introduction

Neuroinflammation and overproduction of proinflammatory cytokines are implicated in the pathological process of chronic neurodegenerative disorders such as Alzheimer's disease (AD) (for recent review see: [1]) and acute trauma such as spinal cord injury (for recent review see: [2]). One of the most well established intracellular signal transduction cascades that regulate the production of proinflammatory cytokines is the p38 mitogen activated protein kinase (MAPK) family [3], [4]. Consisting of four major isoforms (p38α, β, δ, γ), these kinases are encoded by separate genes, expressed in different tissues and cell types, and are often functionally distinct (for review see: [5]). For example, p38α is expressed in most tissues and cell types, whereas the other isoforms are expressed in a more restricted distribution, with p38β high in brain, p38δ in endocrine glands, and p38γ in skeletal muscle [6], [7]. The roles of specific p38 isoforms in various physiological and pathological processes are under active investigation, but available evidence supports the idea that there can be both functional redundancies among the p38 isoforms as well as isoform-specific functions.

In terms of inflammatory pathways, the p38α isoform plays a major role in cytokine up-regulation in both CNS inflammatory disorders and peripheral inflammatory diseases [8], [9]. We previously showed, using both a pharmacological approach with a selective small molecule p38α MAPK inhibitor and a genetic approach with primary microglia that are deficient in p38α, that this isoform is critical for the production of cytokines from activated microglia [8]. Moreover, pharmacological inhibition of p38α MAPK in an AD mouse model decreases brain proinflammatory cytokine production, and attenuates synaptic protein loss [10]. Our more recent study further demonstrated that the deficiency of microglial p38α MAPK rescues neurons and reduces synaptic protein loss via suppressing LPS-induced TNFα overproduction [11]. These data demonstrated that microglia p38α MAPK-mediated cytokine overproduction is critical to inflammation-induced neurotoxicity. However, the role of p38β MAPK in CNS proinflammatory cytokine production and neurotoxicity is poorly understood.

In peripheral inflammatory models, p38β MAPK has been shown to be expendable, with p38β MAPK deficiency affording no protection in arthritis models, inflammatory bowel disease, or LPS-induced TNFα production in the serum [12], [13]. On the other hand, p38β has been reported to be important in protecting mesangial cells from TNFα toxicity, and appears to be involved in the cytoprotective effect of carbon monoxide *in vitro* [14], [15], [16]. Recent studies demonstrated a role for p38β MAPK during sympathetic neuron transdifferentiation [17], as well as reducing sensitivity to pain following spinal cord injury [18], suggesting that p38β MAPK may have specific functions in the CNS that are not seen in the periphery. The goal of the current study was to explore, in a CNS context, whether p38β MAPK signaling contributes to LPS-induced proinflammatory cytokine production, as well as LPS-induced neuronal damage. In agreement with the peripheral inflammatory models, we found that p38β does not play a role in LPS-induced neuroinflammation and neurotoxicity.

## Materials and Methods

### Ethics Statement

All mouse experiments were conducted in accordance with the principles of animal care and experimentation in the Guide For the Care and Use of Laboratory Animals. The Institutional Animal Care and Use Committee of the University of Kentucky approved the use of animals in this study (protocol #2010-0615).

### Reagents

Lipopolysaccharides (LPS) from *Salmonella enterica serotype typhimurium* was obtained from Sigma-Aldrich, St. Louis, MO (Cat. no. L6143-1MG; EU/MG of LPS is 600,000).

### Animals

C57BL/6 mice were obtained from Harlan Laboratories. The p38β MAPK global KO mice were generated [13] and obtained from Dr. Stephen J. O'Keefe at Merck Research Laboratories. Genotyping was performed by Transnetyx, Inc (Cordova, TN).

### Primary neuronal culture

Primary neuronal cultures were derived from embryonic day 18, C57BL/6 mice, as previously described [11]. Briefly, cerebral cortices were dissected and the meninges were removed. Cells were dissociated by trypsinization for 20–25 min at 37°C and triturated, followed by passing through a 70 µm nylon mesh cell strainer. The neurons were plated onto poly-D-lysine-coated 12-mm glass coverslips at a density of 5×10^4^/well in 24 well plates. Neurons were grown in neurobasal medium (Invitrogen) containing 2% B27 supplement (Invitrogen), 0.5 mM L-glutamine (Mediatech), and 100 IU/ml penicillin, 100 µg/ml streptomycin (Mediatech); no serum or mitosis inhibitors were used. Every 3 days, 50% of the media was replenished with fresh medium. The purity of the primary neuronal cultures was verified as 93% by immunocytochemistry for the neuronal marker NeuN, astrocyte marker GFAP, and microglia marker Iba-1 (data not shown).

### Microglia culture

Microglia cultures were prepared as previously described [11]. Briefly, mixed glial cultures (∼95% astrocytes, ∼5% microglia) were prepared from the cerebral cortices of 1–3 day old mice. The tissue was trypsinized as above, and the cells were resuspended in glia complete medium [α-minimum essential medium (α-MEM; Mediatech) supplemented with 10% fetal bovine serum (FBS) (US Characterized FBS; Hyclone; Cat no. SH30071.03), 100 IU/ml penicillin, 100 µg/ml streptomycin, and 2 mM L-Glutamine]. After 10–14 days in culture, microglia were isolated from the mixed glial cultures by the shake-off procedure [19]. Specifically, loosely attached microglia were shaken off in an incubator shaker at 250 rpm for 2 hr at 37°C, the cell-containing medium was centrifuged at 180× *g* for 3 min, and the cells were seeded onto 12-mm glass coverslips at a density of 2×10^4^ in 24 well plates, unless otherwise specified. Prior to plating the microglia on the coverslip, three equally spaced 1 mm glass beads (Borosilicate; Sigma) were attached to the coverslip with paraffin wax. The microglia cultures were verified to be ≥99% microglia by immunocytochemistry. Microglia were incubated for one day before placing into co-culture with neurons.

### Primary microglia/neuron co-culture and cell treatments

After 7 days in culture, neurons on coverslips were co-cultured with mouse microglia as previously described [20], by placing the microglia-containing coverslips cell side down into the neuron-containing wells. In this co-culture system, the microglia and neurons are in close apposition and share the same neurobasal/B27 culture media, but are separated by the 1 mm glass beads and do not have direct cell-cell contact. LPS was resuspended in sterile 0.9% sodium chloride at 100 mg/ml, and was used at a final concentration of 3 ng/ml for all *in vitro* experiments.

### Neuronal Viability Assay

Neuron viability was assayed by trypan blue exclusion [20]. Briefly, neuron-containing coverslips were incubated with 0.2% trypan blue in Hanks' Balanced Salt Solution (HBSS) for 2 min in 37°C incubator and then gently rinsed 3 times with HBSS. Neurons were viewed under bright field microscopy at 200× final magnification. Three to five fields were chosen randomly per coverslip, and a total of 364 to 592 cells were counted per coverslip. Trypan blue-positive and negative neurons were counted per field and the ratio of negative cells to the total cells was taken as the index of neuronal survival rate.

### ELISA assays

After 72 hr in the co-cultures, 20 µl conditioned medium was harvested for TNFα and IL-1β ELISA assay, using kits from Meso Scale Discovery (MSD) according to the manufacturer's instructions.

### In vivo systemic and CNS inflammatory models

Adult mice (3–4 month old, ∼50% male and female) were used for *in vivo* experiments. To induce a systemic innate immune response, mice received intraperitoneal (IP) injection of LPS (1 mg/kg) or sterile saline vehicle in a volume of 100 µl [8]. To induce a CNS inflammatory response, LPS was delivered by intracerebroventricular (ICV) injection. Briefly, mice were anesthetized with 5% isoflurane prior to stabilizing the head using ear bars in digital mouse stereotaxic frame (Stoelting Instruments). Anesthesia was maintained with continuous inhalation of isoflurane (2.5%, 1 liter/min). A midline incision was made in the scalp to expose the skull. A hole was drilled into the skull over the right lateral ventricle at the following coordinates: AP = −0.5 mm; ML = −1.0 mm. Using a 10 µl Hamilton syringe with a blunt 28 gauge needle, saline vehicle or LPS (25 ng) was injected at the following coordinates: AP = −0.5 mm; ML = −1.0 mm; DV = −1.8 mm. The Quintessential Stereotaxic Injector (Stoelting Instruments) was used to inject 2 µl at a rate of 0.5 µl per min. After injection, the needle was left in place for 2 min before being slowly withdrawn. The incision was closed using staples, and the animal was kept on a heat pad until return of normal activity, at which point the mouse was returned to the home cage. At select time points, mice were euthanized with an overdose of sodium pentobarbital (Vibrac Animal Health, cat. no. NDC-051311-103-25), and transcardially perfused with ice-cold phosphate buffered saline (PBS) for 5 min. The brains were rapidly dissected and flash frozen in liquid nitrogen and stored at −80°C for subsequent biochemical evaluation.

### Tissue processing and cytokine measurements

Brain cortex or hippocampus was homogenized using high shear homogenizer (Omni TH115), in a 1∶10 (w/v) of ice-cold freshly prepared lysis buffer consisting of PBS containing 1 µg/ml Leupeptin, 1 mM PMSF, and 1 mM EDTA. The brain lysates were centrifuged at 14,000× *g* for 20 min at 4°C in a microcentrifuge. Fifty microliters of the resulting supernatant (∼100 µg of protein) was loaded per well of the MSD plate, and IL-1β and TNFα levels determined by MSD ELISA assay. Cytokine levels were normalized to the total amount of protein in the sample loaded as determined by BCA Protein Assay (Pierce), and data expressed as pg/ml. The detection limits of the MSD assays are 3.4 pg/ml for TNFα and 1.5 pg/ml for IL-1β.

### Gene expression

RNA was isolated from dissected neocortical tissues stored at −80°C, or from primary microglia cultures using RNeasy mini-columns (Qiagen, Cat no. 74104) with on-column DNase treatment (Qiagen, Cat no. 79254) according to the manufacturer's protocol. RNA quantity and quality were determined using A_260_/A_280_ readings by NanoDrop (Thermo Scientific). Reverse transcription (RT) was done following the manufacturer's protocol using High Capacity cDNA Reverse Transcription Kit (Applied Biosystems, Cat no. 4368814). A no template and a no RT control were conducted to control for contamination. Real-time PCR was performed using the TaqMan Gene Expression assay kit (Applied Biosystems, Cat no. 4444964) according to the manufacturer's instructions on a ViiA™ 7 Real-Time PCR System (Applied Biosystems). The following TaqMan probes (Applied Biosystems) were used: MAPK 11 (Mm00440955_m1); MAPK12 (Mm00443518_m1); MAPK13 (Mm00442488_m1); MAPK 14 (Mm00442507_m1); 18S rRNA (Hs99999901_s1). Relative gene expression was calculated by the 2^−ΔΔCT^ method.

### Statistics

Statistical analysis was conducted using GraphPad prism software V.5 (GraphPad Software, La Jolla, CA). Unless otherwise indicated, values are expressed as mean ± SEM. Groups of two were compared by unpaired t-Test. One-way ANOVA followed by Bonferroni's multiple comparison test was used for comparisons among three or more groups. Statistical significance was defined as p<0.05.

## Results

### Verification of p38β KO in microglia and brain

**Figure 1:**
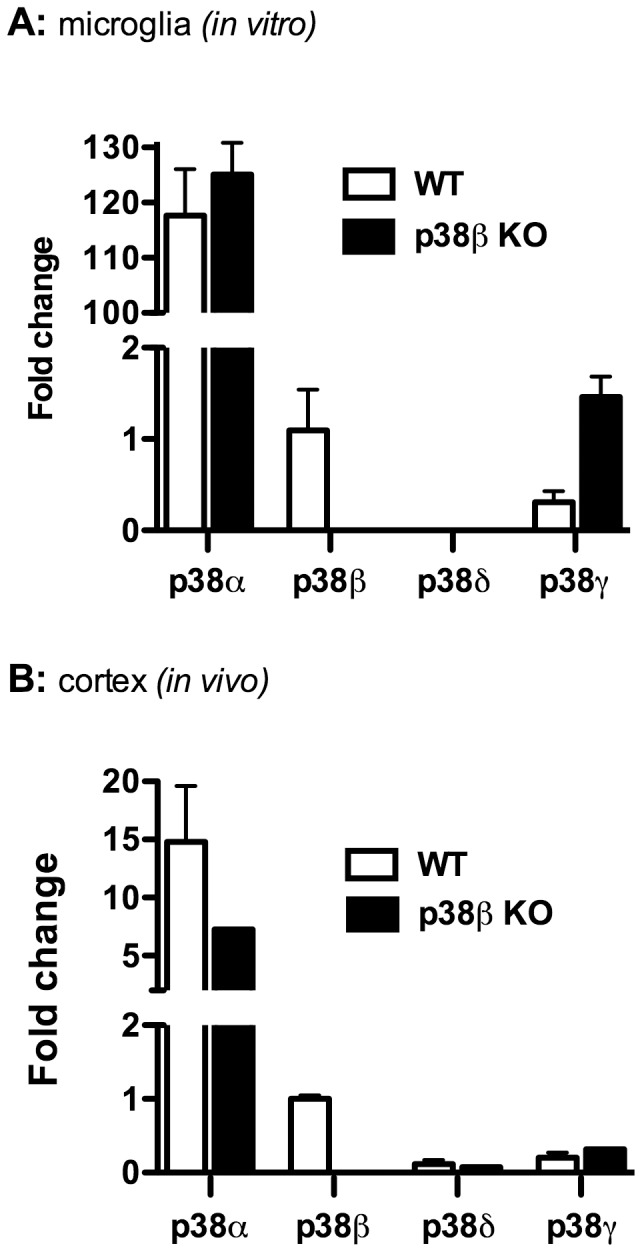
Verification of p38β KO in microglia and brain. Primary microglia from mouse cortex were prepared as described in Methods and plated at 1×10^4^ cells/well in 48 well plates. Total RNA from microglia cultures (**A**) or from mouse cortical tissue (**B**) derived from WT (white bars) or p38β KO (black bars) mice was isolated, and the mRNA levels of different p38 MAPK isoforms were determined by qPCR. In both the microglia cultures and the brain tissues, p38β mRNA was readily measureable in WT mice but was not detected in the p38β KO mice. The p38α MAPK isoform in both microglia and cortex was expressed at much higher levels than any of the other isoforms, and there was no significant difference between the levels of p38α in either WT or p38β KO mice. The levels of p38δ mRNA were very low to undetectable in both WT and p38β KO mice. The expression of p38γ mRNA was slightly higher in microglia cultures from p38β KO mice compared to microglia from WT mice, but this difference was not seen in the cortical tissue samples. Results are expressed as fold change compared to p38β levels, and represent the mean ± SEM of two determinations.

As a control for our studies, it was important to confirm the deletion of p38β MAPK in both the microglia cultures and the mouse brain tissue, and determine that significant compensatory changes in the other p38 isoforms were not present. Therefore, RNA was prepared from primary microglia cultures derived from WT or p38β KO mice (Figure 1A), and from lysates of WT and p38β KO mouse cortical tissue (Figure 1B), and the expression levels of the four p38 MAPK isoforms (p38α, p38β, p38δ, p38γ) were determined by qPCR. In both the microglia cultures and the brain tissue, p38β mRNA was readily measureable in WT mice but was not detected in the p38β KO mice. The mRNA level of p38α MAPK is ∼10–100 fold higher than that of the other isoforms in both WT and p38β KO microglia and brain tissue, and there was no significant difference between the levels of p38α MAPK in either WT or p38β KO mice. The levels of p38δ mRNA were very low to undetectable in both WT and p38β KO mice. The expression of p38γ mRNA was slightly higher in microglia cultures from p38β KO mice compared to microglia from WT mice, but this difference was not seen in the cortical tissue samples. Altogether, these data verify that, as expected, p38β is deficient in microglia cultures and brain tissue from the p38β KO mice.

### p38β KO in microglia did not inhibit LPS-induced cytokine production in microglia/neuron co-culture

**Figure 2:**
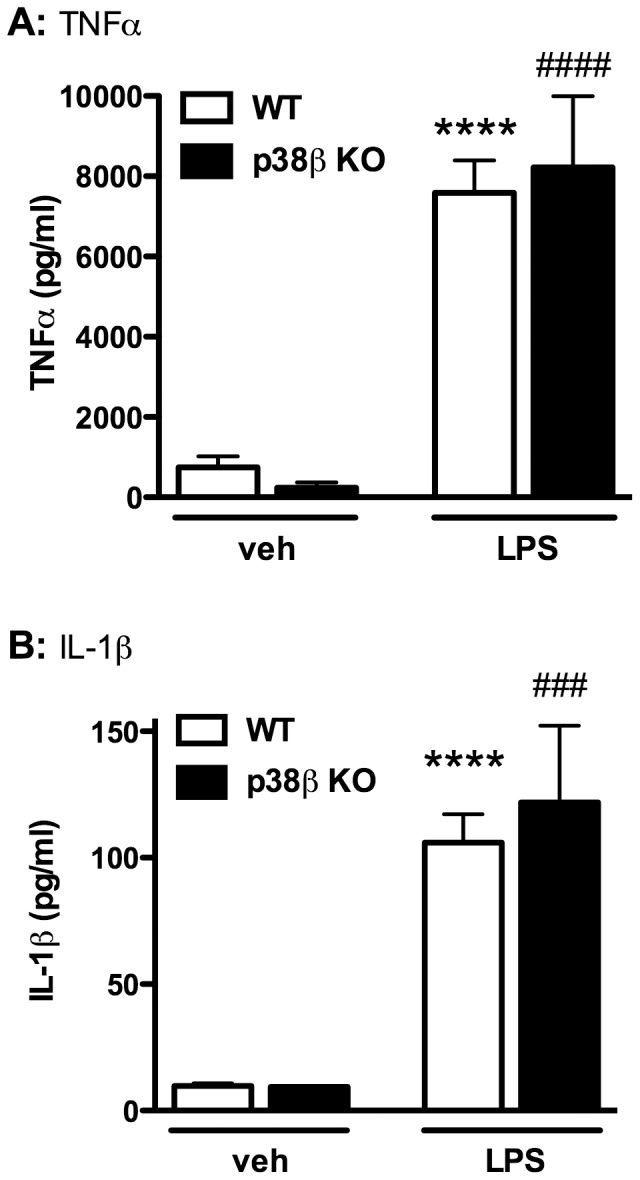
Microglial p38β MAPK deficiency failed to reduce LPS-induced TNFα and IL-1β production in microglia/neuron co-culture. Mouse primary cortical neurons at DIV7 (5×10^4^/well) were co-cultured with microglia (2×10^4^/well) from WT or p38β KO mice, and the cytokine levels of TNFα and IL-1β were measured by ELISA after 72 hr exposure to vehicle or LPS (3 ng/ml). LPS induced a significant increase in TNFα (A) and IL-1β (B) production, and p38β deficiency in microglia did not reduce the production of TNFα and IL-1β. Data represent the mean ± SEM from 2–4 independent experiments. ****p<0.0001 WT-veh vs. WT-LPS; ^###^p<0.001, ^####^p<0.0001 KO-veh vs. KO-LPS.

As an initial step to determine the effect of microglial p38β deficiency on LPS-induced proinflammatory cytokine production, primary microglia from WT or p38β KO mice were co-cultured with WT primary neurons and treated with either vehicle or LPS (3 ng/ml) for 72 hr. LPS induced a highly significant increase in the production of TNFα, and similar levels of TNFα induction were seen in WT or p38β KO microglia (Figure 2A). In a similar fashion, LPS stimulated a ∼100-fold increase in IL-1β levels in both the WT and p38β KO groups, with no differences detected between these two groups upon LPS challenge (Figure 2B). These results document that p38β in microglia is not required for LPS-induced cytokine production.

### p38β KO in microglia failed to protect neurons against LPS-induced neurotoxicity in microglia/neuron co-culture

**Figure 3:**
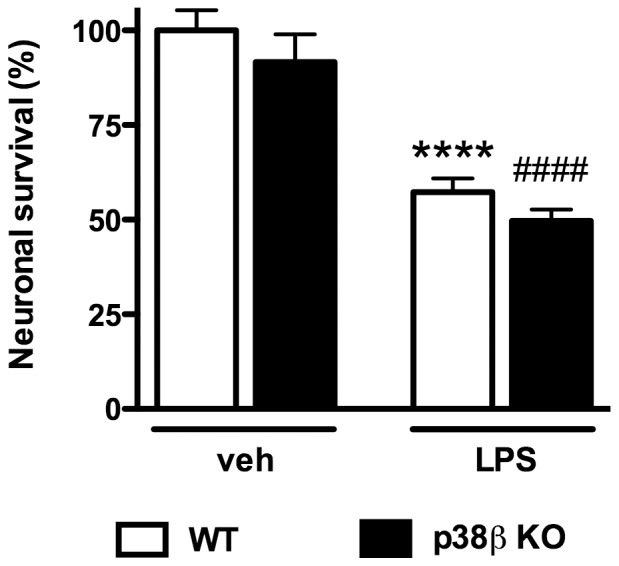
Microglial p38β MAPK deficiency failed to protect cortical neurons against LPS-induced neurotoxicity in microglia/neuron co-culture. WT mouse primary cortical neurons were plated on coverslips at 5×10^4^/well, and were co-cultured with microglia (2×10^4^/well) from WT or p38β KO mice. Cells were treated with either vehicle or LPS (3 ng/ml) for 72 hr, followed by trypan blue exclusion assay to evaluate neuronal survival. LPS treatment induced significant neuronal death in WT microglia/WT neuron co-culture. Similar levels of neuronal death were seen in LPS-treated co-cultures of p38β KO microglia/WT neurons. Data represent the mean ± SEM from 2–4 independent experiments. ****p<0.0001 WT-veh vs. WT-LPS; ^####^p<0.0001 KO-veh vs. KO-LPS.

Previously we found p38α MAPK signaling is important for activated microglia-induced neuronal death in the microglia/neuron co-culture, and that p38α MAPK deficiency in microglia protects against LPS-induced neurotoxicity [11]. However, the potential contribution of microglial p38β MAPK to LPS-induced neurotoxicity is not known. To determine if p38β deletion in microglia can provide similar neuroprotective effects against LPS-induced neuron damage, we isolated microglia from either WT or p38β KO mice, placed them in co-culture with WT primary cortical neurons, and tested whether the absence or presence of microglia p38β would affect LPS-induced neurotoxicity. Treatment of WT microglia/neuron co-cultures with LPS for 72 hr led to significant neuronal death, as determined by trypan blue exclusion assay (Figure 3). In addition, a similar percentage (45%) of LPS-induced neuronal death was found when neurons were co-cultured with p38β KO microglia (Figure 3), showing that p38β MAPK deficiency in microglia does not protect against LPS-induced neurotoxicity.

### p38β KO failed to block CNS proinflammatory cytokine response to systemic and focal LPS insult in vivo

**Figure 4:**
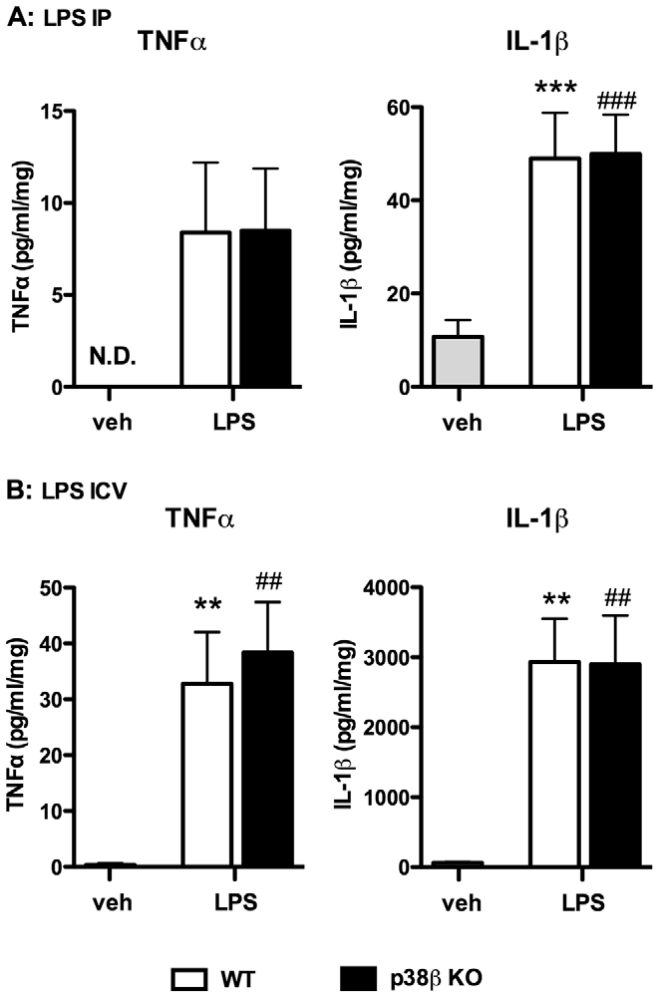
Proinflammatory cytokine levels in WT and p38β KO mouse brain in response to LPS insult. CNS inflammatory responses were induced in WT (white bars) or p38β KO (black bars) mice upon either systemic insult by LPS IP injection (**A**) or direct CNS focal insult by LPS ICV injection (**B**). In the systemic insult paradigm, WT mice and p38β KO mice received IP injection of saline vehicle or LPS (1 mg/kg). Brain samples were harvested at 6 hr post-injection, and the levels of TNFα and IL-1β were determined by ELISA (**A**). At this dose of LPS, the levels of TNFα in the brain were near the level of detection of the ELISA assay, whereas the IL-1β levels showed a significant increase upon LPS insult. The profile of cytokine response to LPS stimulus in p38β KO mice was very similar to the response in WT mice. In the direct CNS focal insult paradigm, saline vehicle or LPS (25 ng) was injected into the right lateral ventricle of WT and p38β KO mice. Brain samples were harvested 6 hr post-injection, and the levels of TNFα and IL-1β were determined by ELISA (**B**). Both TNFα and IL-1β were substantially increased in the brain samples from WT mice upon LPS treatment, and the p38β KO mice showed a similar induction of these proinflammatory cytokines. Results represent the mean ± SEM. Cytokine levels in vehicle-treated mice (gray bars) represent data from both WT and p38β KO mice. n = 6–8 per group for panels A; n = 7–8 per group for panels B. **p<0.01, ***p<0.001 vehicle vs. WT-LPS; ^##^p<0.01, ^###^p<0.001 vehicle vs. KO-LPS. N.D. = not detected.

To test the effect of global p38β deletion on cytokine production in the brain after inflammatory insult, we administered LPS to WT or p38β KO mice by two paradigms: systemic insult by IP injection (Figure 4A), and direct CNS focal insult by ICV injection (Figure 4B).

To induce systemic inflammation, we administered a single dose of LPS (1 mg/kg; IP) to WT and p38β KO mice, harvested cortical tissue at 6 hr after injection, and measured TNFα and IL-1β levels by ELISA. At the dose of LPS used, TNFα levels were near the limits of detection of the assay, but the TNFα levels reached were similar in the WT and p38β KO mice. IL-1β levels increased significantly after LPS treatment, and no significant differences in LPS-induced cytokine levels were seen between the WT and p38β KO mice (Figure 4A).

To determine whether p38β MAPK is important for proinflammatory cytokine production induced by a direct CNS focal inflammation stimulus, LPS was injected into the right lateral ventricle of WT or p38β KO mice. The peak of the IL-1β response in the ipsilateral hippocampus following ICV LPS occurs ∼6 hr post-injection (data not shown); therefore this time point was chosen for cytokine analysis. The result showed that the levels of TNFα and IL-1β increased substantially in both WT and p38β KO mice administered LPS ICV. In addition, consistent with the results from systemic IP administration of LPS, no significant differences in LPS-induced cytokine levels were seen between WT and p38β KO samples (Figure 4B).

## Discussion

In the current study, we used LPS-insulted microglia/neuron co-culture *in vitro* and LPS-induced neuroinflammation in *in vivo* mouse models (IP and ICV) to test a potential contribution of p38β MAPK to the production of proinflammatory cytokines and the survival of cortical neurons. Our results document that p38β MAPK in the brain is not a critical player in LPS-induced neuroinflammation and neurotoxicity, since 1) p38β KO in microglia co-cultured with primary neurons failed to reduce the cytokine production, 2) p38β deletion in microglia cannot rescue neurons against LPS toxicity in co-cultures, and 3) no differences in brain cytokine levels were found between WT and p38β global KO mice in both LPS IP or ICV models. The findings that the LPS-induced CNS responses were not compromised in p38β KO mice support a functionally dispensable role for p38β in LPS-induced neuroinflammation and neurotoxicity. This is in contrast to the critical role of microglial p38α in LPS-induced cytokine production and neuron death in microglia/neuron co-culture [8], [11].

Few studies have examined the role of p38β in CNS disease models, even though this isoform is expressed at substantial levels in the brain [21]. In animal models of ischemia, p38α and p38β levels and activity increased in different cell types and with different temporal kinetics in the post-ischemic brain. For example, in transient global ischemia, p38α was induced early after ischemic insult and was localized mainly to microglia, whereas p38β induction was delayed and more protracted and was localized to astrocytes [22]. In a model of transient focal ischemia, p38β increased early and transiently in neurons, followed by a later increase in astrocytes [23]. Delayed induction of p38β in astrocytes has also been reported in mice subjected to kainate-induced seizures [24], and p38 inhibitor treatment attenuated kainate-induced hippocampal neuron loss and astrocyte activation [25]. Down-regulation of p38β, but not p38α, was also found to reduce sensitivity to pain following spinal cord injury [18]. In contrast to the reports above that suggest a potentially detrimental role of p38β in certain CNS disease models, other studies suggest that p38β may have specific beneficial roles in the CNS. For example, a recent study showed that p38β overexpression provides protection against H_2_O_2_-induced apoptosis in an astrocyte cell line by inducing a small heat shock protein and inhibiting caspase-3 activation [26]. The p38β isoform was also reported to play an important role in the process by which postmitotic sympathetic noradrenergic neurons change to cholinergic neurons, and this neurotransmitter switch mechanism was impaired in neurons from p38β KO mice [17]. Taken together, these previous reports suggest that p38α and p38β may play distinct roles in different CNS disease models, and that their redundant or specific functions may depend on the spatial and temporal features of their activation in response to specific stressors.

The results reported here that p38β does not play a major role in CNS inflammation in response to LPS insult are consistent with previous studies examining the role of p38β in peripheral inflammation models. For example, LPS-induced proinflammatory cytokine production in macrophages and plasma was found to be similar in WT and p38β KO mice, and the p38β KO mice also responded normally in animal models of rheumatoid arthritis and inflammatory bowel disease [12], [13]. These data demonstrated that p38β was not required for these peripheral inflammatory responses. Combined with our findings reported here, the available evidence shows that the p38β isoform does not play a key role in either peripheral or CNS proinflammatory cytokine responses or the resultant neurotoxicity induced by LPS insult. Taken together with our previous studies documenting the critical role of p38α MAPK in mediating LPS-induced CNS proinflammatory cytokine production and neuron survival [8], [11], these results suggest that p38α and not p38β is the key p38 isoform involved in peripheral and central inflammatory responses. These findings also raise the obvious corollary that development of p38 inhibitors to target CNS inflammatory diseases may not need to consider retention of p38β inhibitory activity, but should instead focus on selective targeting of the p38α MAPK isoform as a potential therapeutic strategy.
